# Evidencing strain-dependency of metabolic pathways within 1,494 lactic bacteria genomes with the *in silico* screening Prolipipe pipeline

**DOI:** 10.7717/peerj.21453

**Published:** 2026-07-08

**Authors:** Noé Robert, Jeanne Got, Pauline Hamon-Giraud, Hélène Falentin, Anne Siegel

**Affiliations:** 1 Univ Rennes, Inria, CNRS, IRISA - UMR 6074, Université Rennes I, Rennes, France; 2 Univ Rennes, Inria, CNRS, IRISA - UMR 6074, Centre National de la Recherche Scientifique, Rennes, France; 3 INRAE, Institut Agro, STLO, Rennes, France

**Keywords:** GSM, Bacterial strain dependency, *In silico* pathway screening, Lactic acid bacteria

## Abstract

Genomes from bacteria of interest to the food industry exhibit significant functional variability, yet evaluating this characteristic remains challenging. As public repositories continue to accumulate more genomes, large-scale assessment of metabolic potential emerges as a promising method to highlight this functional variability. The primary challenge lies in automating a workflow to construct metabolic networks from genomes on a massive scale. Here, we present Prolipipe, a pipeline designed for the large-scale assessment of metabolic potential in bacteria, focusing on specific pathways. Given a large dataset of hundreds to thousands of bacterial genomes with known taxonomy and a list of targeted pathways, Prolipipe identifies gene functions through a comprehensive annotation step using three different tools. Then it builds genome-scale metabolic networks for each genome. These networks are then parsed to document the presence or absence of each reaction across all processed genomes. The pipeline evaluates the metabolic potential of each genome to carry out the pathway according to its gene content and highlight the best candidates among the large-scale set of genomes. In this study, Prolipipe was applied to 1,494 genomes of lactic acid bacteria, assessing the completeness ratio of 761 pathways. We classified pathways according to their maximum completeness rate, revealing that 137 pathways can be operated by at least one strain in our dataset. By mapping the identifiers of these pathways onto the pathway ontology graph of the Metacyc database, we highlighted that none of the pathways within four functional classes of Metacyc can be entirely recovered in the strain dataset. We then investigated infraspecific variability, a strong indicator of functional variability, and compared the species in our genome dataset based on their tendency to exhibit infraspecific variability. This analysis revealed species potential for strain-dependency, where phenotypes differ among strains of the same species.

## Introduction

Bacterial genomes of interest to the food industry exhibit a wide range of functional variability, facilitated by mechanisms such as horizontal gene transfer or genomic islands (Stefanovic, Fitzgerald & McAuliffe, 2017). However, precisely determining the functional roles of these genomes and identifying which species exhibit variability remain challenging. To address these questions, public repository databases are increasingly consolidating more and more genomes; the NCBI database (Sayers et al., 2024) features 255,669,865 annotated sequences in GenBank format and 4,152,691,448 sequences from WGS studies as of February 2025. This vast amount of data enables large-scale analyses of metabolic capabilities, including studies aimed at selecting organisms encoding enzymes catalyzing specific reactions of interest. By focusing on the reactions within a given pathway, we can identify organisms capable of addressing specific challenges through their metabolism—either by synthesizing valuable compounds or degrading unwanted metabolites. The latter approach requires linking a specific pathway to its reactions, the enzymes catalyzing them, their genes, and identifiers—information stored in databases such as KEGG (Kanehisa et al., 2025; Kanehisa, 2019; Kanehisa & Goto, 2000) or MetaCyc (Caspi et al., 2020).

Ultimately, the greatest challenge lies in the large-scale analysis of potential metabolic capabilities using tools designed to construct genome-scale metabolic networks (GSMs). While several solutions exist for building GSMs—such as Bactabolize (Vezina et al., 2023) which relies on ModelSEED (Seaver et al., 2021) and AutoKEGGRec (Karlsen, Schulz & Almaas, 2018), very few are scalable to hundreds of genomes while delivering results at a metabolic pathway scale. Converting annotated genomes into metabolic data demands extensive computational parallelization, a task that tools like Mpwt (Belcour et al., 2020) or CarveMe (Machado et al., 2018) can accomplish. Working with large datasets enables the identification of metabolic specificities but requires strictly standardized processing of raw data to prevent annotation biases or inconsistencies arising from non-homogeneous datasets. Moreover, it is not possible to reason about pathways with compound-specific accuracy using databases such as BIGG (King et al., 2016)—on which CarveMe relies- or ModelSEED (Seaver et al., 2021), since these either do not include pathways or present them at a scale too broad to be compound-specific. To address these challenges, we present Prolipipe (AuReMe, 2025), a tool for large-scale metabolic profiling of bacteria, focusing on specific pathways. Its strategy is based on raw genomes as input, which are processed through a robust annotation step, followed by targeted and standardized metabolic network construction. To evaluate its scalability and accuracy, Prolipipe was applied to a dataset of 1,494 bacterial genomes, analyzing 761 MetaCyc pathways. The results revealed infraspecific variability in strains of the same species, demonstrating Prolipipe’s capacity to uncover strain-specific metabolic capabilities.

## Methods

Portions of this text were previously published as part of a preprint (https://hal.science/hal-05045657v4/document)’.

### Creation of a large catalogue of bacterial genomes

A dataset of lactic acid bacteria’s genomes (LAB) is built in compliance with the following restrictions: the genomes had to be qualified as presumptively safe (QPS) according to the EFSA agreement (EFSA, 2022), display no ability for sporulation, or have any known pathogenic effects on plants. As a result, 1,494 LAB genomes were retrieved from the NCBI FTP server in the fna FASTA format. Taxon and strain files were built to complete Prolipipe’s inputs by linking species names and taxonomic identifier to files and strain name and status to files, respectively. The list of considered strains is available at https://zenodo.org/records/15351076.

### Selection and creation of Prolipipe-compatible pathways files

The MetaCyc database (version 27.0) references a total of 3,489 pathways. From these, we selected 1,493 pathways classified as “observed in bacteria” to ensure our analysis focused on reactions catalyzed by enzymes with documented evidence in bacterial genomes. Since pathways with two or fewer reactions—comprising half of the initial set—can introduce bias into downstream metrics (such as pathway completeness, which depends on the number of reactions), they were excluded from the study. This filtering process resulted in a final set of 761 pathways for analysis.

### Prolipipe, a glance of the workflow

**Figure 1:**
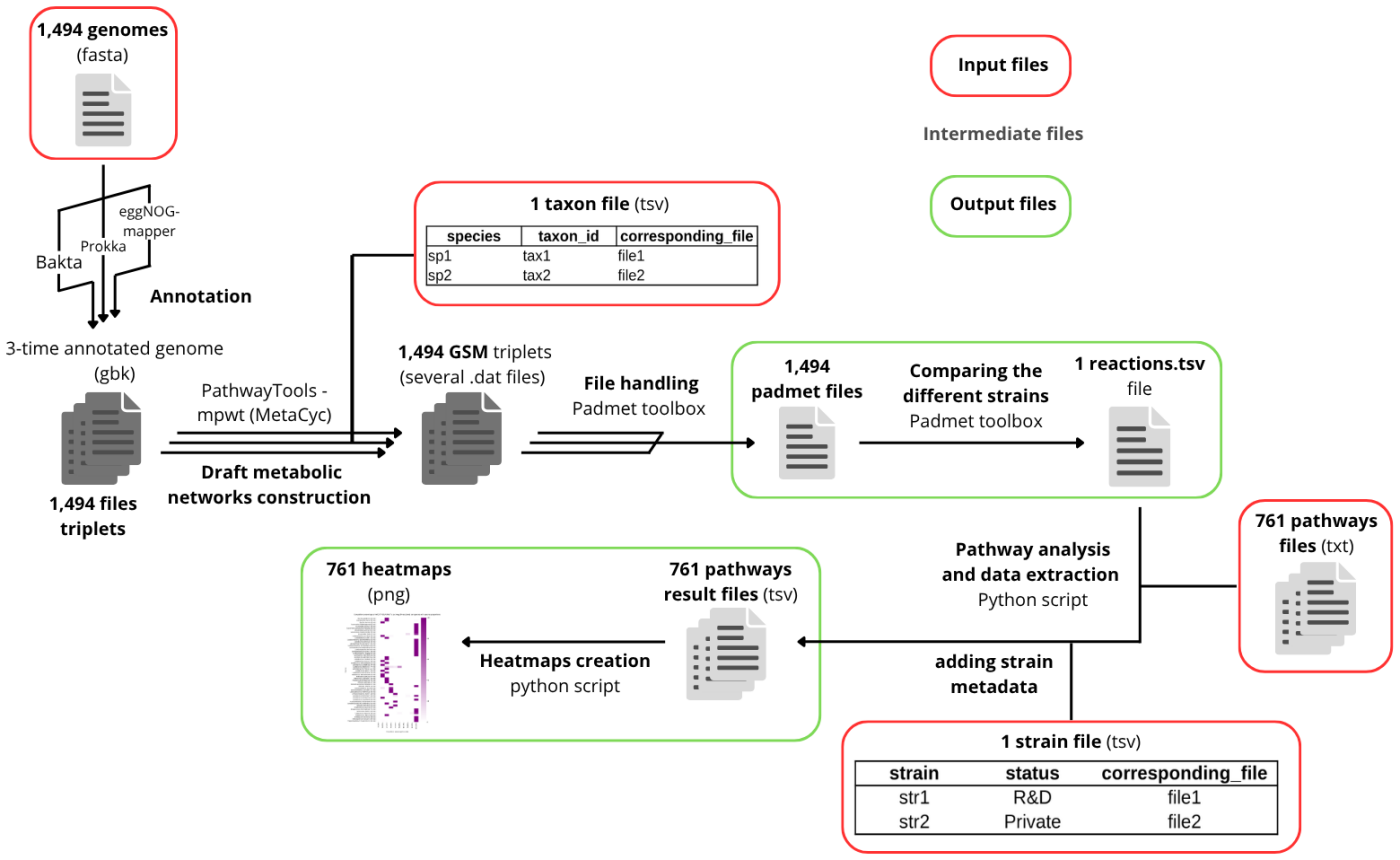
Prolipipe application to our dataset. 1,494 genomes along with a taxon file, a strain file and 761 metabolic pathways were processed using Prolipipe, which first annotated the genomes before constructing their genome-scale metabolic networks (GSM). These GSMs were then aggregated into a database and queried to assess the metabolic ability of each strain for each given pathway; the results were stored in pathway-specific tables which were subsequently used to generate heatmaps.

We implemented Prolipipe, a Python package whose steps are summarized in Fig. 1. Given a large number of genomes along with a taxon file, a strain file and a list of metabolic pathways, the first step of Prolipipe consists of genome annotation using a homogeneous structural and functional annotation approach. This step relies on three tools that have demonstrated their individual role in gene annotation: Prokka version-1.14.6 (Seemann, 2014) using the Prokka database (version 20/02/2023) with the—*compliant* flag; EggNOG-mapper v2.1.12 (Cantalapiedra et al., 2021) relying on the eggNOG 5.0 database (Huerta-Cepas et al., 2019) and using the—*itype genome* and—*genepred prodigal* options; Bakta 1.8.2 (Schwengers et al., 2021) with its own database (version 20/02/2023). Each of these tools generates output files in GenBank format, resulting in three annotation versions per strain. The second step consists of generating draft genome-scale metabolic networks (GSM). To achieve this, GBK files are processed *via* a parallelized application of the PathoLogic algorithm from Pathway Tools (Karp et al., 2021) using the Python package Mpwt (Belcour et al., 2020). The previously generated taxon file is provided to refine the analysis, and the *–patho* flag is used to allow PathoLogic inference and the integration of all reactions from the MetaCyc database. At this stage, draft GSMs (three per genome) contain sets of .dat files, the native format of Pathways Tools. These files are managed through the PADMet toolbox (Aite et al., 2018), a Python package offering a comprehensive suite of tools for metabolic pathway reconstruction and annotated genome comparison. For this study, we used the *pgdb_to_padmet* command along with the *–extract-gene*, *–no-orphan*, and *–source* options for downstream benchmarking, as well as *–padmetRef* option to retain only reactions associated with gene sequences from the draft GSMs and spontaneous reactions. This command generates a single .padmet file per strain and annotation tool. Finally, padmet files corresponding to the same strain are concatenated to create a consensus GSM per strain using the *padmet_to_padmet* command. During this step, there is no competition between annotation sources, as all reactions obtained from the three sources are integrated.

### Prolipipe’s GSM post-analysis and metabolic networks database generation

GSMs are then queried to assess the metabolic profile of each strain based on the input pathways. Prolipipe compiles a database from all GSMs described within the Padmet files, enabling further comparisons using the *compare_padmet* command from the PADMet toolbox (Aite et al., 2018). All results are aggregated into four files, including one named reactions.tsv which documents the presence or absence of reactions across all processed strains. This file is analyzed by Prolipipe to produce pathway-specific result tables, which are then used to generate heatmaps that highlight strain dependency. Alternatively, Prolipipe can produce output files compatible with a SPARQL-endpoint, facilitating the generation of a queryable database. The pipeline is available on GitHub (https://github.com/AuReMe/prolipipe).

## Results

### Benchmarking of annotation tools used

**Figure 2:**
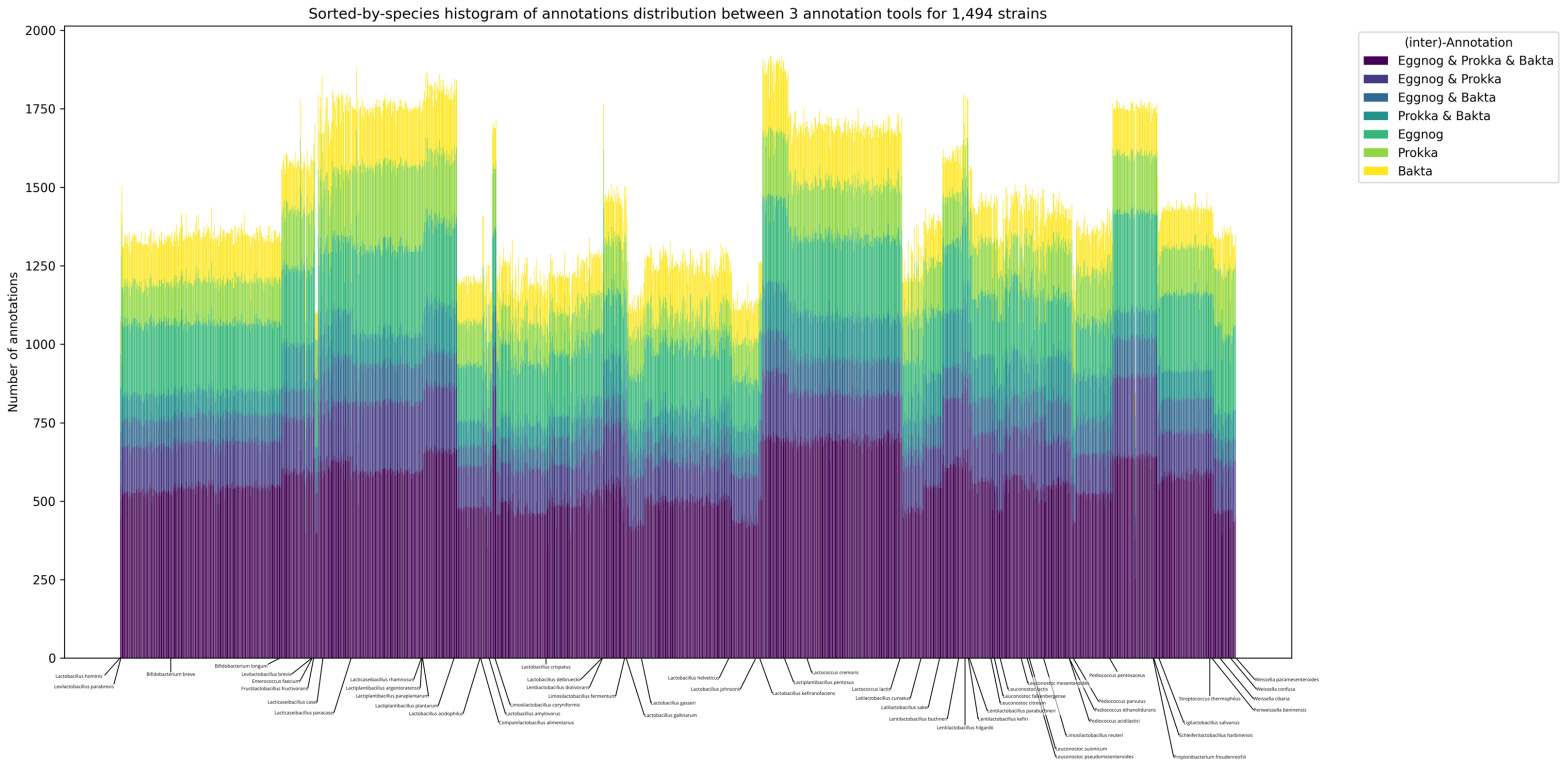
Annotation tools benchmark by evaluating the number of annotations from each tool combination within all 1,494 strains. Species are labelled on their last individual. Annotations of enzymes that resulted in a MetaCyc reaction identifier are obtained by PathwayTools (*via* mpwt) from EC numbers and GO terms.

Prolipipe, which records the annotation source when annotating genomes using EggNOG-mapper, Prokka, and Bakta, was run on 32 CPUs for 171 h to construct a catalog of 1,494 bacterial genome-scale metabolic models (GSMs). The GSMs were sorted by species, with the number of reactions per model ranging from 845 to 1,918, as shown in Fig. 2. The maximum variance observed originates from the species *Propionibacterium freudenreichii*, where the minimum number of reactions detected in genomes is notably low (845 reactions). This variation in the number of identified reactions results in a maximum standard deviation of 117 among strains of the same species, reflecting heterogeneity in the number of annotations derived from the three annotation sources. The bar chart illustrates the contribution of the three annotation tools across all 1,494 GSMs. We observed that the three annotation tools generate consensus predictions accounting for 30 to 43% of the size of the GSMs. Additionally, our analysis highlights a distinct distribution of annotation confidence levels: consensual annotations (predicted by all three tools), strongly supported annotations (predicted by two tools), and tool-specific annotations (predicted by only one tool). Tool-specific annotations represent 10 to 16% of all reactions in GSMs. Within this category, Bakta-specific annotations show a significant enrichment in Enzyme Commission (EC) numbers linked to hydrolases acting on halide bonds (EC 3.8.-.-) and ether bonds (EC 3.3.-.-). EggNOG annotations, in turn, are enriched in cysteine-type carboxypeptidases (EC 3.4.18.-) and oxidoreductases acting on other nitrogenous compounds as donors (EC 1.7.-.-). Prokka annotations, meanwhile, exhibit enrichment in endoribonucleases producing products other than 5′-phosphomonoesters (EC 3.1.27.-) and oxidoreductases acting on hydrogen as donors (EC 1.12.-.-).

### Maximum completeness rate per pathway

Prolipipe pipeline was executed on 1,494 bacterial genomes to assess the completeness rate of 761 bacterial metabolic pathways for each genome. This rate is calculated as the ratio of reactions within the pathway linked to a gene, as identified by at least one annotation tool, to the total number of reactions in the pathway.

**Figure 3:**
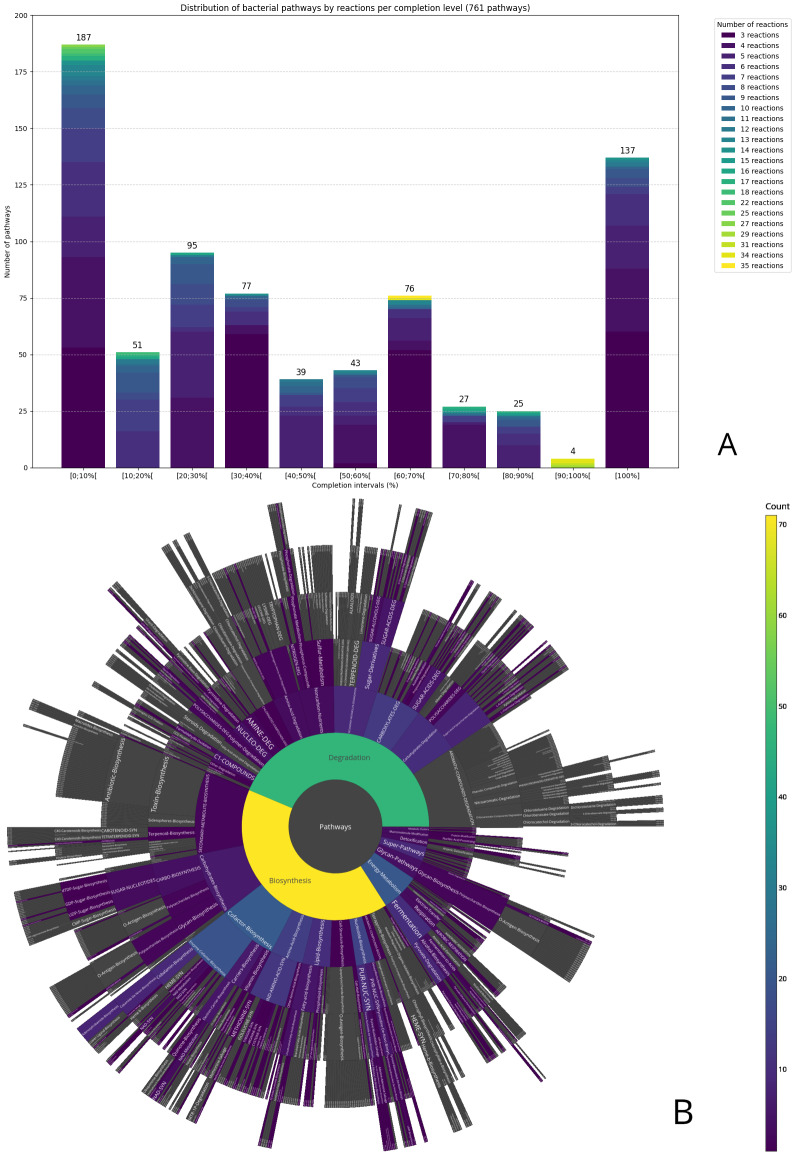
(A) Bar chart of pathway completeness rate distribution. 761 pathways are dealt depending on their completeness rate which is splitted into 10%-completeness brackets, with an eleventh column for strictly complete pathways. The number of reactions per pathway is indicated with the shade of color. (B) Sunburst diagram on pathways ontology. 137 pathways are found completed by at least one of the dataset’s strain and their repartition in the MetaCyc ontology is screened on all the 761 processed pathways, with colored areas having at least one of these completed pathways. The colorbar used shows headcounts in classes; that way, colored classes show which pathways can be completed by the dataset’s strains.

The bar chart in Fig. 3A illustrates the distribution of pathways according to their maximums completeness rate, grouped into 10% completeness intervals, with a dedicated column for pathways achieving 100% completeness. Since placement within the intervals depends heavily on the number of reactions in each pathway, bars are color-coded accordingly. A total of 187 pathways exhibited low completeness rates below 10%. This corresponds with the fact that the 1,494 genomes catalogue is composed of lactic acid bacteria which are known for their specialized metabolic properties and inability to perform all known metabolic pathways. Conversely, 137 pathways achieved full completeness in at least one strain, as indicated by the (100%) interval column. Furthermore, 29 pathways exhibited a completeness between 80% and 100%, prompting further analysis. Among them, 10 pathways contained at least one reaction lacking an affiliated EC identifier.

Focusing on the 137 pathways completed by at least by one strain, the sunburst diagram from Fig. 3B illustrates their distribution within the Metacyc database ontology for all 761 bacterial pathways. Ontology classes shown in color indicate the presence of at least one fully completed within that class, such as galactose degradation (MetaCyc ID: PWY-6317). This pathway aligns with the expected metabolic profile of lactic acid bacteria LAB—known for being able to catabolize this sugar present in milk—used in this study (Wang et al., 2021). Prolipipe’s table output reveals that 1,384 strains out of 1,494 contain all reactions necessary for this pathway. In contrast, ontology classes depicted in gray indicate categories with no fully completed pathways. These include toxin biosynthesis, degradation of aromatic compounds, lipopolysaccharide synthesis and O-antigen biosynthesis.

### Infraspecific variability assessment

**Figure 4:**
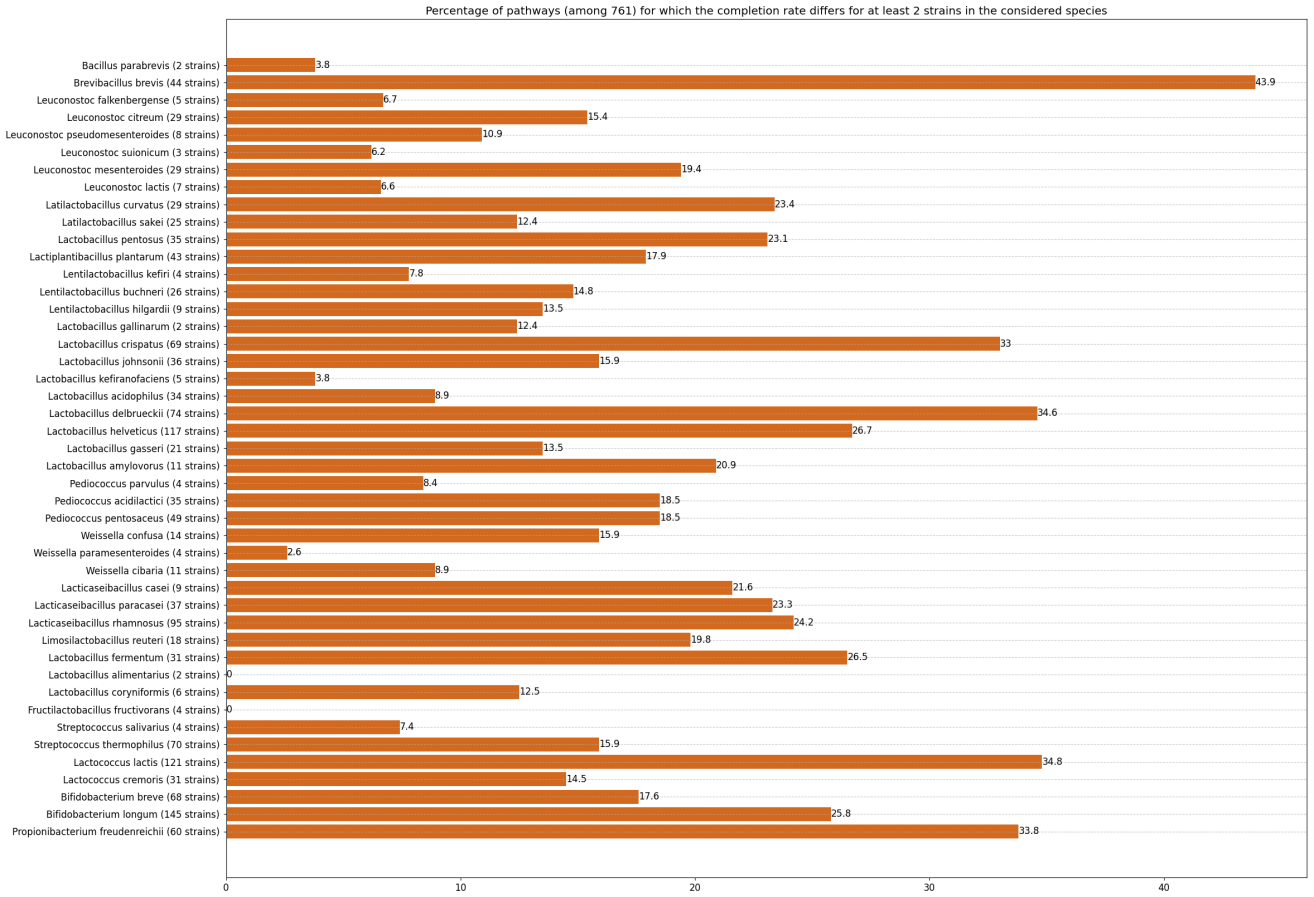
Bar-chart of infraspecific variability frequency. Each species represented by more than one individual is represented by a bar, with the horizontal axis indicating the ratio of observed infraspecific variability across all 761 analyzed pathways. Infraspecific variability is identified when two strains within a species exhibit completeness rates for a given pathway that differ by at least 10%. This suggests that the strains in question have different enzymatic capabilities associated with the metabolic pathway.

Given a pathway, differences in completeness rates between strains of the same species indicate the potential for infraspecific metabolic variability. These differences reflect variations in genes annotation related to a specific pathway among strains of the same species. By aggregating data derived from pathway completion heatmaps, we can directly assess infraspecific metabolic variability: since these heatmaps calculate the percentage of strains falling within specific completion brackets, the absence of a “100” value in a given row indicates variability in pathway completion within the same species (*i.e.,* the “100” value is distributed across multiple columns). To quantify this phenomenon, we counted the occurrence of such events and normalized the result by the total number of pathways analyzed. The use of 10% brackets is justified by the average size of the pathways studied—5.6 reactions—and the fact that only 9% of pathways contain 10 or more reactions. For pathways of this size, 10%-completion brackets are appropriate, as a more precise scope would not significantly affect the majority of pathways. The bar chart depicted in Fig. 4 obtained that way shows the percentage of infraspecific variability observed over 761 pathways per species (45 species represented by 1,485 strains of the catalogue have more than 1 individual, out of 54 species). This percentage ranges from 0 to 43.9% of all pathways with on one hand *Brevibacillus brevis* showing 334 pathways out of 761, suggesting intra-species metabolic variability. On the other hand *Bifidobacterium longum* shows less variability with a ratio of 25.8% (196 out of 761) while being the most represented species with 145 individuals. This example demonstrates that, although species are not equally represented in the genome catalog, differences in intra-species metabolic variability potential can still be detected. However, it is important to note that species representation in our dataset is uneven, and sampling bias may arise from underrepresented species; their counts are provided in the figure. Infraspecific variability may occur when only a subset of a species’ strains complete a given pathway, resulting in phenotypic differences among these strains. This phenomenon, referred to as strain dependency, is discussed in further detail below.

### Statistical test on the correlation between assembly quality and downstream completion score

The dataset comprises genomes assembled at various levels of completeness: Contig (444), Scaffold (570), Chromosome (1), Complete Genome (443), and unknown (36). Given that differences in assembly quality may influence intraspecific diversity due to potential sequence gaps, we investigated the relationship between pathway completion rates and genome assembly quality. Genomes were categorized into two groups: “assembled” (complete genomes and chromosomes) and “draft” (scaffolds, contigs, and unknown assemblies). For each metabolic pathway, we calculated the number of genomes achieving the highest completion rates and assessed their distribution across the two assembly quality classes. To determine whether genome assembly quality affects pathway completion, we performed a Pearson’s Chi-Square test for each pathway, with the null hypothesis (H_0_) stating no relationship between assembly quality and optimal completion scores. A significance threshold of 0.05 was applied. Out of the 761 pathways initially tested, we excluded those with uniform results—specifically, pathways lacking any detectable reactions in the dataset, where all genomes were trivially classified as having the “best completion score”. Among the remaining 583 pathways, statistical analysis revealed that 398 pathways exhibited completion rates independent of assembly quality, *i.e.,* 68% of pathways. Conversely, 185 pathways demonstrated a significant correlation between genome assembly quality and optimal pathway completion rates. To avoid conclusion on false positives, we applied FDR correction on all the 583 Chi-Square tests’ *p*-values, which raised the number of pathways showed with their completion rate independent of assembly quality from 398 to 439, reaching 75% of analyzed pathways. As N50 is a good indicator of genome quality, we applied the same method to study independence between N50 values and completion scores. We performed a Pearson’s Chi-Square for each pathway, with the null hypothesis (H_0_) positing no relationship between N50 value (whether it is above 1,500,000 bp (484 genomes) or below it (1,010 genomes)) and optimal completion scores. The same significance threshold of 0.05 was applied, and among the 583 Chi-square tests performed on the 761 considered pathways (the remaining having uniform data), 457 of them (78%) retained H_0_ after FDR *p*-value correction and showed independence between N50 value and the ability to perform the best pathway completion score.

### Focusing on L-arginine biosynthesis through acetyl cycle

**Figure 5:**
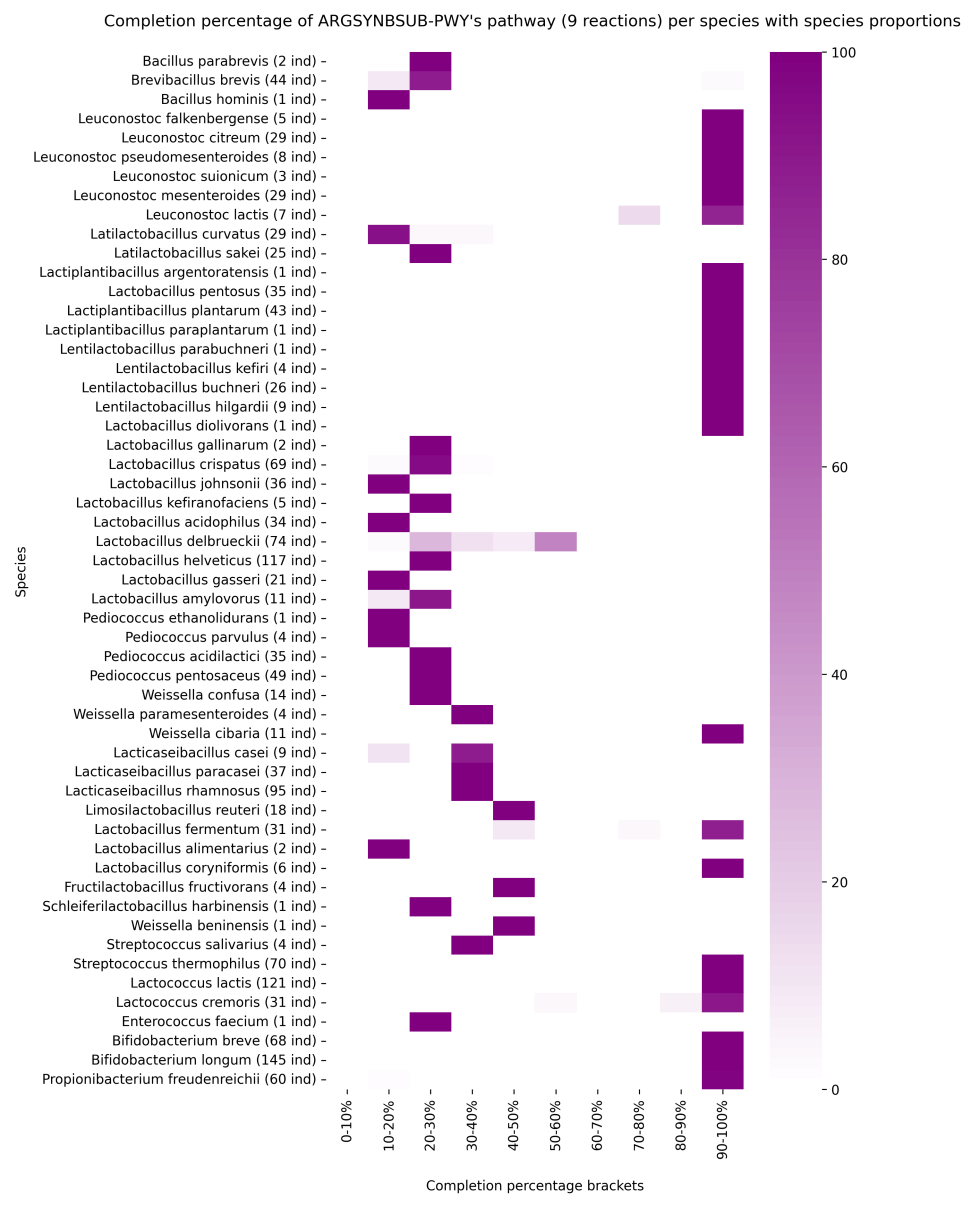
Completeness heatmap of ARGSYNBSUB-PWY (Metacyc ID of L-arginine biosynthesis pathway through acetyl cycle) within all 1,494 genomes. Completeness ratio among the 54 species (represented in line, headcount given in label) is dealt on 10%-completion brackets, proportion of individuals inside a species in shades of purple.

The heatmap in Fig. 5 depicts the completeness rate of the L-arginine biosynthesis through the acetyl cycle, a metabolic pathway enabling organisms to produce L-arginine from L-glutamine and L-glutamate through nine different reactions. completeness ratios were obtained for the 1,494 strains (covering 54 species) of our dataset using Prolipipe. The *X*-axis divides completeness percentage values into 10%-intervals, strains are ordered taxonomically and grouped into the 54 species of the dataset, and color shade represents the proportion of strains within a species whose completeness rate falls within a specific interval.

A total of 23 species exhibited a fully colored cell in the 100% completeness column, indicating that all their representative strains achieved full pathway completeness. These species are strong candidates for carrying out the pathway and producing L-arginine under suitable environmental conditions. For example, *Streptococcus thermophilus* (70 strains) achieved 100% completeness, confirming its status of candidate for carrying out the pathway entirely.

In contrast, 28 species did not contain any strain with 100% completeness rate. Among these, some exhibited high variability in completeness rates. *Lactobacillus delbrueckii*, for instance, displayed five different completeness rates represented by several cells of different shades on a same line: two strains have genomes annotated with the genes of one reaction; 21 strains are associated with two reactions; nine strains are associated with three reactions; six strains are associated with four reactions and 36 strains are associated with five reactions. For these 28 species, the ability to synthesize L-arginine through the acetyl cycle is considered unlikely. As this pattern may reflect database gaps rather than genuine biological absence, we sought to distinguish between these two possibilities by focusing on reactions that were never present in the entire set of GSMs. The 2,278 never-observed reactions account for 54% of the 4,191 reactions represented across the 761 bacterial pathways. Notably, only 45% of these never-observed reactions (1,017) possess a complete Enzyme Commission (EC) number (four digits separated by periods), compared to 93% of the remaining 1,913 reactions. A Chi-Square test confirmed a significant association between the absence of a reaction and the validity of its EC number.

Finally, three species—*Leuconostoc lactis*, *Lactobacillus fermentum* and *Lactococcus cremoris*- displayed infraspecific variability. In *Leuconostoc lactis*, six out of seven strains (85.7%) achieved 100% completeness. Similarly, in *Lactobacillus fermentum* and *Lactococcus cremoris*, 87.1% and 90% of strains, respectively, reached full completeness. These findings indicate that most strains within these species have the genetic potential to synthesize L-arginine through the acetyl cycle. However, other strains associated with these species have low completeness rates. This suggests that the considered species are candidate to be strain-dependent with respect to the phenotype of arginine production (Bringel & Hubert, 2003).

## Discussion

From the previous figures, we demonstrated that Prolipipe has significant potential for studying large-scale datasets of bacterial genomes and enables two key analyses. The first key analysis is a large-scale detection of bacterial strains which are promising candidates for the effective production or degradation of metabolites based on their genome content. In this context, Prolipipe can be used for genome mining by identifying bacteria with the highest potential for pathways of interest, such as biosynthesis of target compounds or degradation pathways. This process is facilitated by the use of sunburst diagrams that utilize pathway ontology to identify over- and under-represented functional classes. An example was provided with galactose degradation, where Prolipipe output indicated that this pathway is predominantly present in our strains’ genomes, which corroborates the literature (Wang et al., 2021) and demonstrates its compatibility with genome mining approaches.

A second key analysis is the detection of infraspecific variability in pathway completeness rate within large-scale genome catalogues. This analysis can be extended to comparison between species, provided that the dataset is homogeneous in terms of species representation. Indeed, in our current catalogue, species headcounts range from one individual to 145, thereby complicating the discrimination between true infraspecific variability, known to exist within LAB (Thierry et al., 2015) and biases arising from unequal species representation. Nevertheless, these two aspects—the detection of accessible pathways and infraspecific variability—are directly visualized in readily available, pathway-specific heatmaps which serve as valuable tools for more detailed studies of metabolic profiles related to a given metabolic pathway within a bacterial dataset. Moreover, such displays can reveal candidates for strain-dependency, defined as differences in pathway completeness rate among strains of the same species, with a subset of the specie’s strains achieving 100% completeness of the pathway. It is important to note that, since this analysis is based solely on genomic data, the associated phenotypic traits must be validated experimentally.

Prolipipe’s annotation step integrates three complementary annotation methods, each significantly enhancing the identification of metabolic reactions within GSM models. By leveraging all three state-of-the-art methods with the concatenation of the reactions contained in padmets obtained from these three sources, the pipeline ensures a comprehensive and robust annotation process. The primary objective of Prolipipe is to efficiently screen large genomic datasets, prioritizing the reduction of candidate genomes to a manageable subset—even at the risk of introducing false positives. Since each annotation tool relies on distinct sequence databases for gene identification, the use of multiple tools mitigates potential biases that could arise from database-specific enrichments. Additionally, because Prolipipe tracks the origin of all gene-reaction associations, it enables the ranking of pathways based on the number of annotation tools supporting each gene association, thereby enhancing confidence in functional predictions. Finally, we aim to enhance the metabolic reconstruction process by employing dedicated function predictors for genome annotation and by implementing a consensus annotation system based on confidence scores. This approach will enable the selection of high-confidence signals according to these scores.

We identified three main sources of false negatives—cases where a pathway’s completion score fails to reach 100% in a strain’s GSM, despite the strain’s demonstrated ability to perform the relevant function.

First, false negatives may arise from poor genome assembly quality where gaps in the sequence can prevent the annotation of genes responsible for synthesizing the enzymes involved. While our dataset exhibits heterogeneity in assembly quality, potentially affecting enzyme annotation counts, prior statistical analyses revealed that 78% of pathways showed independence between N50 values and the ability to achieve optimal completion scores. This suggests that, although the risk of false negatives due to assembly quality exists, it is relatively low. Selecting genomes with higher assembly quality can further reduce this risk, but it remains an important consideration when interpreting metrics such as intraspecific variability.

Second, false negatives may result from gaps in the metabolic database. Some reactions lack sufficient genomic data to be inferred from annotated genomes, often due to missing EC numbers linking annotation to metabolic data. This limitation persists even with the inclusion of spontaneous reactions in metabolic network construction. A Chi-Square test confirmed a dependency between the absence of a reaction and the validity of its EC number. Since Prolipipe relies on MetaCyc—a database known for annotation biases favoring aerobic and pathogenic bacteria—such gaps are inevitable. However, by analyzing large-scale genomic catalogs, Prolipipe can flag these gaps as anomalies. For example, the systematic absence of a reaction, despite extensive genomic datasets, may indicate incomplete functional annotations or gene-reaction associations rather than a true biological absence. To address this, Prolipipe uses an “adjusted completion rate” metric, which excludes undetected enzymes and focuses on identifying candidate strains.. The main limitation of this approach is its reliance on gene-reaction associations (linked *via* EC and MetaCyc identifiers). The previously quoted tool suite Padmet, which is part of the workflow, allows enrichment of the database with reactions coming from other sources, although the following has not been tested yet. The development of specialized annotation tools targeting enzymes presents a promising solution to this bottleneck, and Prolipipe is designed to integrate such tools to improve completeness.

Third, false negatives can occur when an unknown pathway, not present in the database, is responsible for the strain’s phenotype. For these rare cases in bacteria, there is currently no solution to bypass this limitation.

Additionally, there is a risk of false positives due to the triple annotation process, where pseudogenes might be misidentified as functional genes. This risk can be mitigated by adjusting annotation tool parameters and increasing the stringency of gene coverage criteria. However, given these potential errors, Prolipipe should be viewed as a preliminary tool for assessing diversity potential.

Prolipipe’s capabilities are currently being extended to metagenomics, where metabolic capability assessment would not be limited to individual organisms but would encompass entire bacterial communities, facilitating research on metabolic complementarity. This extension is expected to be valuable for the development of bacterial consortia to address challenges that single strains cannot overcome. Additionally, Prolipipe is being adapted for eukaryotic analysis, specifically for assessing metabolic machineries in yeasts.

## Conclusion

In this paper, we presented Prolipipe and demonstrated its capacity to perform two main types of analyses. First, as originally designed, Prolipipe enables genome mining by identifying the most promising candidate strains from large genomic datasets to calculate the completeness rate of specific pathway. Second, it detects infraspecific variability in pathway completeness rates within extensive genome catalogs, though its accuracy can be influenced by factors such as uneven species representation. Our study identified three primary sources of false negatives: genome assembly quality, gaps in knowledge databases, and incorrect pathway analysis. While these factors did not significantly affect our results, they must be considered, and we have proposed strategies to mitigate or assess their impact where possible. For these reasons, Prolipipe should be viewed as a preliminary tool: its main purpose is to filter out less promising strains, not to ensure quality or comprehensiveness. Ultimately, validating Prolipipe’s predictions requires *in vitro* experimentations, such as controlled fermentations in bioreactors by inoculation of most promising strains identified by Prolipipe. This tool will open new avenues in term of metagenomics and microbial consortia analysis. Finally, it will be extended to eukaryotic organisms analyses.

## Additional Information and Declarations

### Data Availability

The following information was supplied regarding data availability: The data, including all strains used from NCBI, is available at Zenodo: Robert, N. (2025). Prolipipe on Lactic Acid Bacteria: strain’s genomes metadata (1.0) [Data set]. Zenodo. https://doi.org/10.5281/zenodo.15351076
The code is available at GitHub and Zenodo: - https://github.com/AuReMe/prolipipe
- Robert, N. (2025). Prolipipe code. Journées Ouvertes en Biologie, Informatique et Mathématiques (JOBIM), Bordeaux. Zenodo. https://doi.org/10.5281/zenodo.18374414
